# Truncated mass divergence in a Mott metal

**DOI:** 10.1073/pnas.2301456120

**Published:** 2023-09-11

**Authors:** Konstantin Semeniuk, Hui Chang, Jordan Baglo, Sven Friedemann, Stanley W. Tozer, William A. Coniglio, Monika B. Gamża, Pascal Reiss, Patricia Alireza, Inge Leermakers, Alix McCollam, Audrey D. Grockowiak, F. Malte Grosche

**Affiliations:** ^a^Cavendish Laboratory, University of Cambridge, Cambridge CB3 0HE, United Kingdom; ^b^Max Planck Institute for Chemical Physics of Solids, Dresden 01187, Germany; ^c^Department of Physics, Université de Sherbrooke, Sherbrooke J1K 2R1, Canada; ^d^H H Wills Laboratory, University of Bristol, Bristol BS8 1TL, United Kingdom; ^e^National High Magnetic Field Laboratory, Tallahassee, FL 83810; ^f^Jeremiah Horrocks Institute for Mathematics, Physics and Astronomy, University of Central Lancashire, Preston PR1 2HE, United Kingdom; ^g^Max Planck Institute for Solid State Research, Stuttgart 70569, Germany; ^h^High Field Magnet Laboratory, Radboud University, Nijmegen 6525 ED, The Netherlands; ^i^Leibniz Institute for Solid State and Materials Research, IFW Dresden, 01069 Dresden, Germany

**Keywords:** Mott localization, quantum oscillations, high-pressure techniques

## Abstract

The Mott metal–insulator transition represents one of the most fundamental phenomena in condensed matter physics. Yet, basic tenets of the canonical Brinkman-Rice picture of Mott localization remain to be tested experimentally by quantum oscillation measurements that directly probe the quasiparticle Fermi surface and effective mass. By extending this technique to high pressure, we have examined the metallic state on the threshold of Mott localization in clean, undoped crystals of NiS_2_. We find that i) on approaching Mott localization, the quasiparticle mass is strongly enhanced, whereas the Fermi surface remains essentially unchanged; ii) the quasiparticle mass closely follows the divergent form predicted theoretically, establishing charge carrier slowdown as the driver for the metal–insulator transition; iii) this mass divergence is truncated by the metal–insulator transition, placing the Mott critical point inside the insulating section of the phase diagram. The inaccessibility of the Mott critical point in NiS_2_ parallels findings at the threshold of ferromagnetism in clean metallic systems, in which criticality at low temperature is almost universally interrupted by first-order transitions or novel emergent phases such as incommensurate magnetic order or unconventional superconductivity.

## Significance

Strong repulsive interactions in metals can cause electronic grid-lock, producing a Mott insulator. The strongly correlated metallic state on the brink of such Mott localization is associated with some of the most challenging emergent phenomena in condensed matter research. This study tracks the electronic charge carrier concentration and velocity in a clean three-dimensional material tuned toward the Mott insulating state by applied pressure. Our results show that carriers slow down dramatically as the Mott transition is approached, confirming experimentally the Brinkman–Rice description of the correlated Mott metal, first articulated more than fifty years ago. These findings inform the study of more complex materials such as cuprate, organic, and iron-based superconductors, Moiré superlattice systems, and Kondo lattice materials.

Mott localization is one of the most fundamental consequences of electronic interactions in solids (1). Its theoretical understanding feeds into numerous related research areas, ranging from cuprate superconductivity to organic conductors, Moiré superlattice systems, and correlated topological insulators (2–5). The Mott insulating state (6) is stabilized near half-filling, when the on-site repulsion energy U exceeds a threshold value $U_{c}$. In the simplest case laid out in the Hubbard model (7), this threshold is determined by the kinetic energy contribution to the total energy. Various factors further affect $U_{c}$, for instance, charge transfer into additional bands near the Fermi energy or more than one half-filled state per lattice site and the resulting Hund’s coupling. Although quantum materials of current interest often require more elaborate models that reflect, for instance, the interplay of slow and fast carriers in Kondo lattice systems, key aspects of these materials connect back to the Hubbard model, such as the notion of orbitally selective Mott transitions in multiband systems (8–11).

**Fig. 1.**
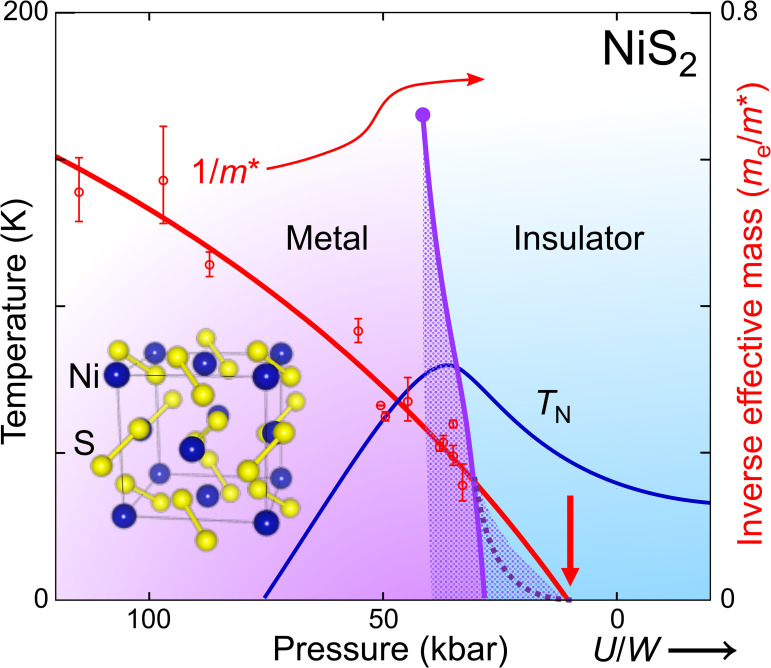
Mott metal–insulator transition in NiS_2_, which is metallic at high pressure (large effective bandwidth W and therefore small ratio U/W) and insulating at low pressure. Magnetic order sets in below a transition temperature $T_{N}$ [blue line, following experimental data (19, 20)]. The Mott transition line [purple, from resistivity data (21)] ends in a critical point at high temperature. Its first-order nature implies the possibility of metastable states (dotted region) surrounding the thermodynamic transition line. At low temperature, the transition line in DMFT calculations curves away toward higher U/W (dashed purple line), ending in a zero-temperature critical point (16–18) (thick red arrow). The inverse carrier mass extracted in this study (red circles with errorbars) extrapolates to zero deep inside the region of the phase diagram where transport measurements show insulating behavior.

In the canonical description formulated by Brinkman and Rice (12), Mott localization is driven not by reduction of charge carrier concentration (as is the case in band insulators) but rather by a gradual slowing down of the charge carriers, while the volume enclosed by the Fermi surface remains constant in line with Luttinger’s theorem (13). In this description, the reduction of the Fermi velocity of strongly correlated Landau quasiparticles is reflected in a reduction of the quasiparticle weight z toward zero and a concomitant rise and, ultimately, divergence of the quasiparticle effective mass $m^{*}$. More sophisticated calculations within dynamic mean field theory (DMFT) have supported this scenario for the evolution of the correlated metallic state in the low-temperature limit for a purely electronic Mott transition (14–18). As illustrated in Fig. 1, these calculations indicate that the transition is first order at finite temperature, that it is accompanied by a range in which metallic and insulating states can coexist (dotted region in Fig. 1) and that the transition line bends sharply as the zero temperature limit is approached (dashed line in Fig. 1).

Given the central importance of Mott physics for understanding quantum materials, it may be surprising that experimental tests of the Brinkman–Rice paradigm are comparatively scarce. Spectroscopic measurements such as photoemission spectroscopy (PES) (22, 23) examine the suppression of the quasiparticle weight in the strongly correlated metal near Mott localization, and they can track the spectrum close to and well away from the Fermi energy (24). However, the limited energy resolution of PES and its inability to distinguish between the coherent and incoherent parts of the spectrumhinder high precision measurements of the coherent low-energy excitations that constitute long-lived Landau quasiparticles, as is apparent for instance in ref. 25. PES also tends to be limited to elevated temperatures, and it cannot be undertaken under pressure, which presents a clean way to tune in small steps across a Mott transition without doping-induced disorder.

Conversely, the Landau quasiparticles at the core of the Brinkman–Rice picture are detected directly by observing quantum oscillatory phenomena in high magnetic fields. These probe the quasiparticle spectrum, making it possible to track the quasiparticle mass and Fermi surface as Mott localization is approached with superior resolution. Such measurements have contributed to reports of a divergent form of the quasiparticle mass at the magnetic quantum critical points in CeRhIn${}_{5}$ and BaFe${}_{2}$(As${}_{1-x}$P${}_{x}$)${}_{2}$ (26, 27). Quantum oscillation studies under applied pressure have probed the evolution of the correlated metallic state near Mott localization in 2D organic charge transfer salts (28, 29), but because of the wider pressure range required, significant technical challenges have to be overcome to apply this methodology in a 3D inorganic Mott system.

Here, we study the clean 3D Mott insulator NiS_2_ and use high-pressure quantum oscillation measurements as a direct probe of the coherent quasiparticles and their Fermi velocity. Our data confirm key tenets of the Brinkman–Rice model, namely that charge carrier concentration is conserved but carrier mass takes on a divergent form on approaching Mott localization. At the same time, we find that the carrier mass divergence extrapolates to a critical point which is buried well inside the insulating state and is apparently inaccessible. The resultant picture of Mott localization is reminiscent of the scenario occurring in clean metallic systems at the threshold of magnetic order, where quantum criticality is almost universally interrupted by first-order transitions, tricritical behavior, or novel emergent phases such as unconventional superconductivity (30–32).

The cubic sulphide NiS_2_ offers an excellent opportunity to investigate the correlated Mott metal, because high purity single crystals with negligible vacancy concentration are available (20, 33) and a moderate pressure of about 30 kbar is sufficient to reach the metallic state (20, 21, 34, 35) (Fig. 1). A previous study demonstrated that quantum oscillations can be observed under these conditions (21). Avoiding doping and the associated disorder, this affords a direct view on the evolution of quasiparticle properties within the correlated metallic state.

**Fig. 2.**
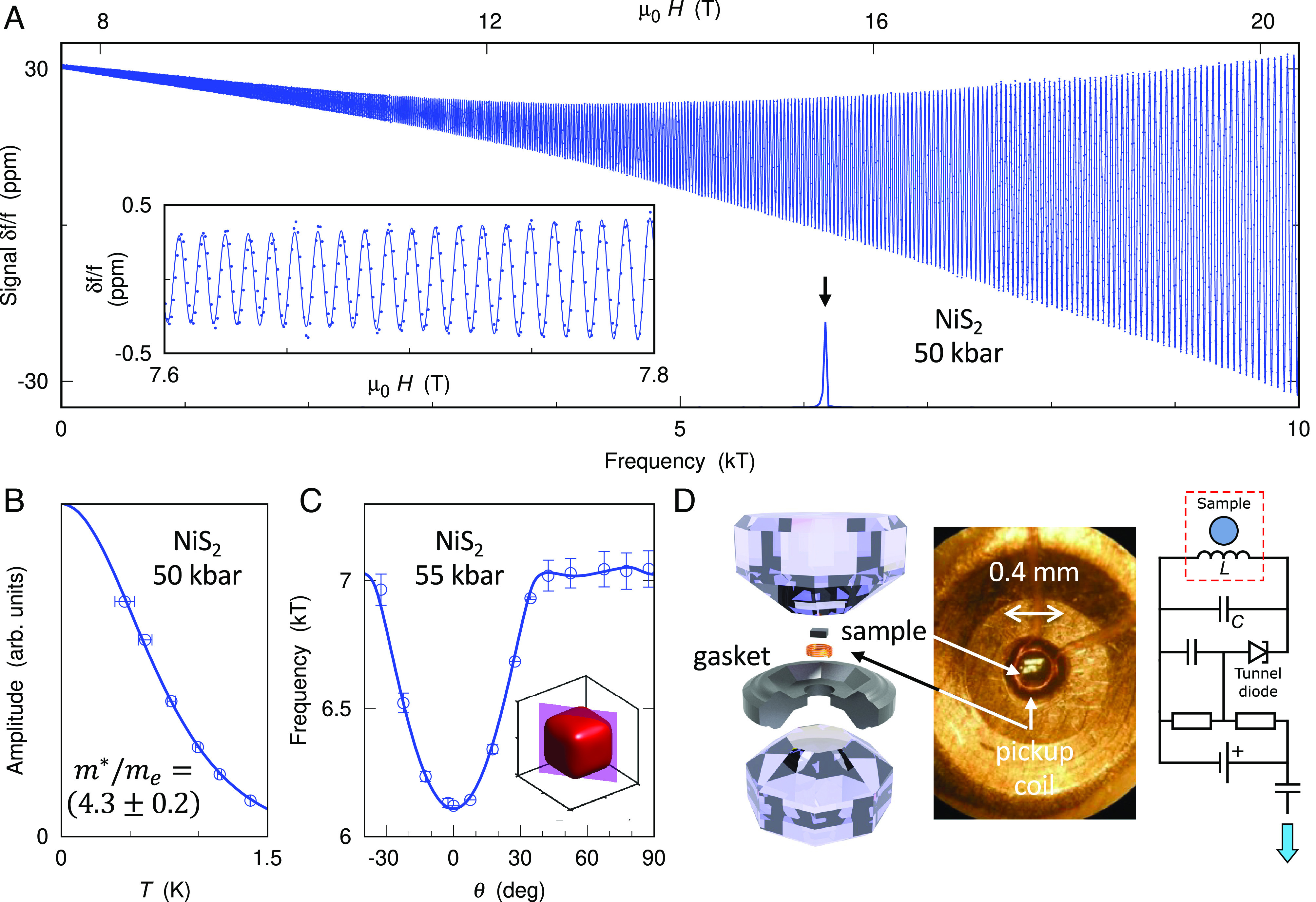
Quantum oscillations in NiS_2_. (*A*) Quantum oscillations are clearly resolved well above the metallization pressure, down to fields as low as <8T (*Inset*). The power spectrum shows a single peak at 6.17 kT (*Lower* axis). (*B* and *C*) The QO amplitude (*B*) follows the Lifshitz–Kosevich form as a function of temperature (solid line, see *SI Appendix* for the relevant details), with effective mass $m^{*}≃4.3\;m_{e}$, and a rotation study (*C*) at a nearby pressure produces an angle dependence of the QO frequency that closely matches expectations (solid line) for a cube-shaped FS pocket (inset). (*D*) Key elements of the experimental setup.

The detection of quantum oscillations under pressure in NiS_2_ (21) builds on three innovations: i) Te-flux growth (33) reliably produces high-quality crystals matching the best vapor-transport grown NiS_2_ reported previously (20), with residual resistivities of ≃1μΩ cm at high pressure (21); ii) tank circuit radio frequency tunnel diode oscillator (TDO) techniques (36, 37) with a microcoil placed inside the <400 μm diameter high-pressure sample space enable ultrasensitive, contact-free measurements of skin depth or magnetic susceptibility oscillations in applied field at high pressures (Fig. 2*D*); iii) miniature anvil cells and novel gasket preparation techniques allow access to the 100 kbar range and beyond in narrow-bore dilution refrigerator probes or on rotator stages while preserving excellent pressure homogeneity (38). Combined with superheterodyne signal detection, such a setup can resolve quantum oscillations at the level of 0.01 ppm. Additional details concerning the sample preparation and experimental techniques are provided in (*SI Appendix*).

## High-Pressure Quantum Oscillations.

**Fig. 3.**
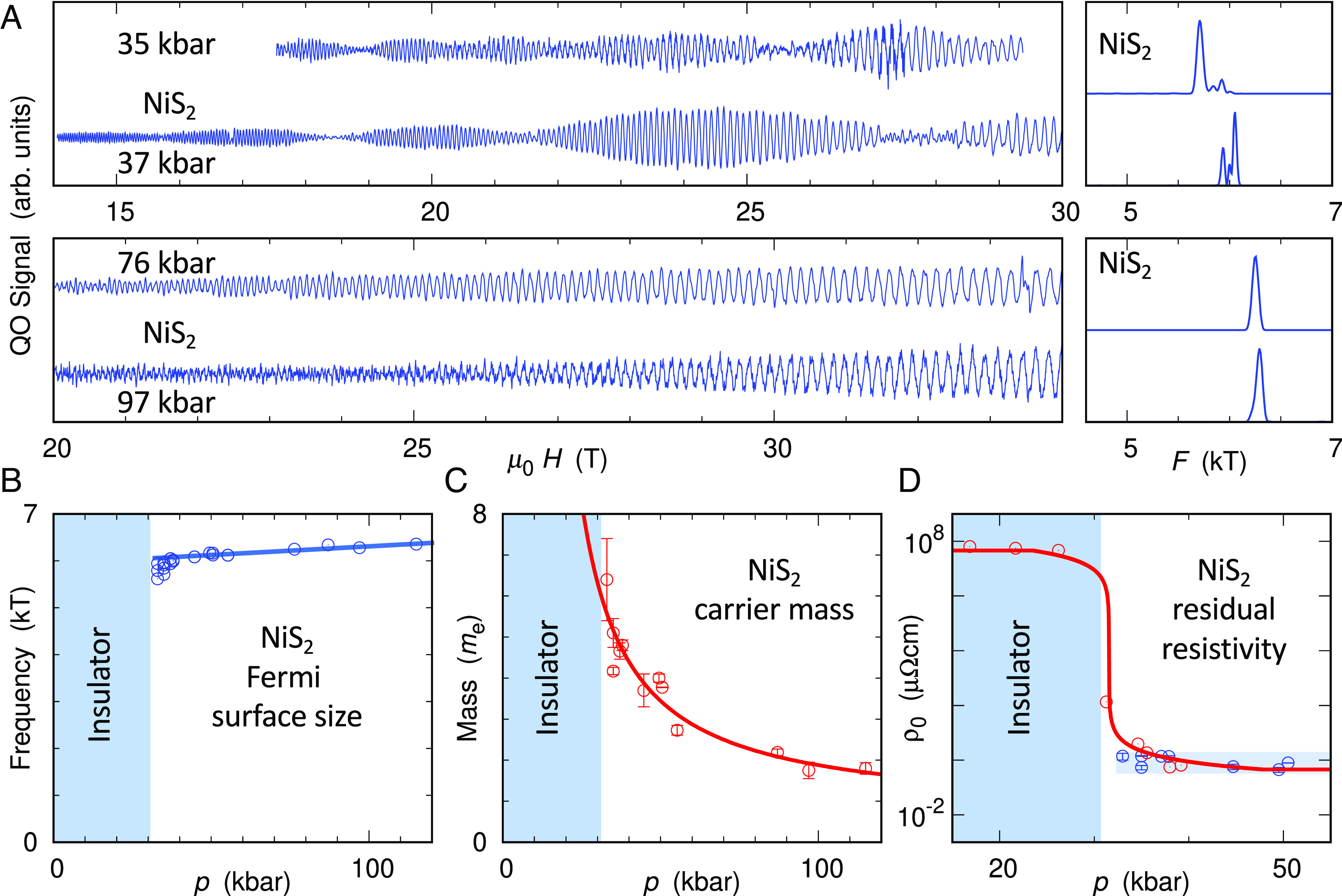
Pressure evolution of the electronic structure of NiS_2_. (*A*) Quantum oscillations in NiS_2_ at selected pressures (scaled and off-set for clarity), with corresponding power spectra to the right. (*B*–*D*) Pressure dependences of the quantum oscillation frequency (*B*), the effective quasiparticle mass (*C*) and the residual resistivity $\rho_{0}$ (d). The blue line in panel (*B*) is a linear least-squares fit to the frequency coming from the cubic Fermi surface (the highest frequency at each pressure). The red line in panel (*C*) is a least-squares fit consistent with the one shown in Fig. 1 (see text). Although the effective mass grows by more than a factor of three over the investigated pressure range, the frequency and thereby the Fermi surface cross-section changes only slightly, at a rate which is broadly consistent with the change of the unit cell volume. Panel (*D*) compares $\rho_{0}$ determined in high-pressure transport measurements (21) (red markers) to $\rho_{0}$ expected from the electronic mean free paths determined from the quantum oscillation analysis (blue markers, the analysis is explained in the *SI Appendix*). Although the quantum-oscillation-derived mean free path and the resulting $\rho_{0}$ are pressure independent (blue shaded region), direct transport measurements show a strong increase of $\rho_{0}$ on approaching the metal–insulator threshold from high pressures. This discrepancy suggests a significant and increasing volume fraction of insulating regions close to the metal–insulator threshold, which we model within 3D effective medium theory (solid red line, explained in the *SI Appendix*). We ascribe the saturation of the transport-derived $\rho_{0}$ below 30 kbar to surface conduction (42).

The Fermi surface signatures detected in our quantum oscillation measurements in NiS_2_ closely match density functional theory (DFT) calculations for the largest Fermi surface sheet (see *SI Appendix* for the DFT predictions). We observe strong quantum oscillations with a frequency F≃6.17kT as illustrated in (Fig. 2) at a pressure of 50 kbar, well above the metallization pressure of 30 kbar. This frequency is in close agreement with our earlier measurements at 38 kbar and with the accompanying DFT calculations (21). It corresponds to a cross-sectional area $A_{k}$=0.589Å${}^{-2}$ for B||c, nearly half the cross-sectional area of the first Brillouin zone (BZ) $A_{\mathrm{BZ}}$=1.27Å${}^{-2}$. The angular dependence of the quantum oscillation frequency closely matches expectations from a cube-shaped Fermi surface pocket (Fig. 2*C*). A hole pocket of almost identical size and shape is the dominant feature in ab initio DFT calculations within the paramagnetic metallic state (21), confirming the assignment of these quantum oscillations to the cube-shaped hole surface. A small splitting of the quantum oscillation frequency at lower pressures (Fig. 3 *A*, *Top*) can be attributed to the effect of magnetic ordering (39) (*SI Appendix*). Although ab initio DFT calculations can accurately reflect the Fermi surface geometry, they capture electronic correlations insufficiently to produce reliable estimates of the true carrier mass, which can be detected directly in quantum oscillation measurements.

The carrier mass is strongly renormalized. The effective quasiparticle mass $m^{*}=4.3\;m_{e}$ ($m_{e}$ - free electron mass) determined at 50 kbar from the temperature dependence of the oscillation amplitude (Fig. 2*B*) exceeds the ab initio band mass $m_{b}=0.8\;m_{e}$ obtained for this hole pocket by a factor of 5.4, indicating substantial mass renormalization comparable to the highest values observed by quantum oscillation measurements in any transition-metal compound (40, 41) (the analysis of the quantum oscillations is described in *SI Appendix*). This highlights the strongly correlated nature of the metallic state in NiS_2_.

The Fermi surface volume is preserved over the full pressure range, indicating that the charge carrier concentration remains constant on approaching the metal–insulator transition. The pressure dependence of the quantum oscillation frequency and carrier mass, based on 14 high-pressure runs reaching up to ∼ 115 kbar, are summarized in Fig. 3. The pressure dependence of the quantum oscillation frequency can be attributed to the compressibility of NiS_2_ as indicated by the solid line in Fig. 3*b*. We use the change of unit cell volume determined from X-ray diffraction to estimate the effect of pressure on the Fermi-surface volume (data provided in *SI Appendix*). Transport measurements in the Ni(S/Se)${}_{2}$ composition series indicate increases in resistivity and Hall coefficient on approaching the insulating threshold by reducing the Se content, which could be interpreted in terms of a decreasing carrier concentration (43). Our data show that this scenario does not apply in pressure-metallized NiS_2_: the nearly constant quantum oscillation frequencies observed right up to the insulating state demonstrate that no significant change in the carrier concentration is taking place. We note that quantum oscillations directly probe the Fermi surface volume in reciprocal space, whereas contrary inferences from Hall effect measurements (43) may suffer from the effects of inhomogeneities and magnetic contributions.

The carrier mass is strongly boosted on approaching the Mott metal–insulator transition, suggesting critical slowing down of the coherent quasiparticles. We observe a monotonic increase of the quantum oscillation mass between 120 kbar and ≃30 kbar (Fig. 3*C*), ruling out magnetic quantum criticality associated with the threshold of antiferromagnetism at ≈80 kbar (44) as the primary driver of the mass enhancement. The effective mass of the coherent quasiparticles in NiS_2_ grows by more than a factor of three over the pressure range investigated, translating to a seven-fold enhancement over the bare (DFT) band mass, which in our calculations shows negligible pressure dependence over the range investigated experimentally.

## Mott Critical Point Concealed within the Insulating Phase.

While the carrier mass follows a divergent form consistent with the Brinkman–Rice scenario over a wide range of pressure, the critical point for the mass divergence is buried within the insulating part of the phase diagram. The quasiparticle mass is expected to diverge as $1/(p-p_{c})$ within the Brinkman–Rice picture close to a critical pressure $p_{c}$. More generally, the inverse mass enhancement given in ref. 12 follows $m_{b}$$/m^{*}=1-{\left(U/U_{0}\right)}^{2}$, where $U_{0}$ is proportional to the bandwidth and can be expanded to first order in pressure p: $U_{0}=A\left(p+a\right)$, with constant coefficients A and a. At the critical pressure $p_{c}$, $U_{0}=A\left(p_{c}+a\right)=U$, leading to the simpler expression for the inverse mass renormalization $m_{b}$$/m^{*}=1-{\left(\left(p_{c}+a\right)/\left(p+a\right)\right)}^{2}$. This expression closely fits the pressure dependence of the quantum oscillation mass (Fig. 3*C*), with a critical pressure $p_{c}≃10\pm 1\;\;\text{kbar}$ well below the metallization pressure of about 30 kbar determined from transport experiments (21). The inverse mass enhancement, likewise, is shown in Fig. 1 to extrapolate to zero inside the insulating regime.

The resulting phase diagram (Fig. 1) is consistent with DMFT studies for a purely electronic Mott transition (14–18). As mentioned in the introduction, the first-order thermodynamic transition line in these calculations bends sharply toward higher coupling at low T, and it is surrounded by a region in which both metallic and insulating states coexist, one being thermodynamically stable, the other metastable. Below the pressure of the high T critical point, $p_{h}≃44\;\;\text{kbar}$, the low-temperature state is accessed by cooling through the transition line which overhangs the metallic region of the phase diagram. If the thermodynamic transition line is crossed at sufficiently high temperature, enough of the material converts into the metallic state to enable the observation of quantum oscillations. The reduction in QO signal amplitude and the rise in $\rho_{0}$ on approaching the metal–insulator transition in NiS_2_ (21) (Fig. 3*D*) as well as Ni(S/Se)${}_{2}$ (43) suggest that the metallic volume fraction is already strongly reduced well before the metal–insulator transition itself is reached. The reduction in metallic volume fraction also explains why the bulk Sommerfeld ratio C/T in the Ni(S/Se)${}_{2}$ decreases slightly on approaching the MIT (43). In this purely electronically driven scenario, the metallic volume fraction diminishes as the second-order low-temperature end point of the Mott transition is approached, truncating the observed mass divergence. Coupling to the lattice, which causes a discontinuous volume change, can further exacerbate the first-order nature of the MIT and extend it to zero temperature (45), folding away the section of the pressure–temperature phase diagram surrounding the second-order low-temperature end point of the transition line, which thereby becomes inaccessible to experiment.

This interpretation is consistent with high-pressure x-ray data, which reported evidence for phase coexistence near metallization in NiS_2_ (46), with ARPES data in Ni(S/Se)${}_{2}$, which indicates that the Fermi velocity $v_{F}$ extrapolates to zero within the insulating part of the phase diagram (24, 25), and with high-pressure heat capacity measurements in V${}_{2}$O${}_{3}$, in which the divergence of the Sommerfeld coefficient does not occur at the metal–insulator transition but rather can be extrapolated well into the insulating phase (47).

In summary, our high-pressure quantum oscillation measurements allow the first comprehensive survey of the electronic structure and its evolution on the metallic side of a pressure-induced Mott insulator transition in ultraclean specimens with a simple crystal structure. A large Fermi surface pocket is detected in pressure-metallized NiS_2_, which corresponds closely to expectations from band structure calculations. On approaching Mott localization from the metallic side, the Fermi surface volume remains comparatively unaffected right up until metallic behavior is lost. The carrier mass, on the other hand, rises in a clean, divergent form with unprecedented dynamic range for correlation-induced mass renormalization.

Our results complement the central tenets of the Brinkman–Rice picture with the realization that in this cubic system, the expected mass divergence on approaching Mott localization is truncated by the consequences of a first-order phase transition, reminiscent of the fate of magnetic quantum critical points in metallic magnets (30). Resolving the precise origin of this phenomenon may be a challenge for future experiments which could, for instance, approach the critical pressure from the metallic side at low temperature in a variable-pressure device. Our results furthermore demonstrate the power of anvil cell–based quantum oscillation measurements extending into the >100 kbar regime for addressing challenging questions in fundamental condensed matter research. This provides a powerful complement to ARPES studies, which are limited to ambient conditions, and opens up a wide range of long-standing problems for closer investigation. The metallic state on the threshold of a Mott insulator transition is a central research theme in modern condensed matter physics. Its relevance is born out by the intense effort devoted to the normal state of the high-temperature superconducting cuprates. Additional complexity arises from coupling to the lattice (45, 48), orbital degeneracy and Hund’s rule coupling (24, 49, 50), phase separation and percolation (51), colossal susceptibility to applied electric field or strain (52–54), and novel electronic surface or bulk states (55). The methodology developed for this study may help investigate these and other challenging phenomena that arise in the correlated metallic state on the threshold of Mott localization—or more generally near pressure-induced quantum phase transitions.

## Materials and Methods

### Synthesis of NiS_2_ Single Crystals.

Single-crystal samples were grown with the tellurium-flux method as described earlier (33). Ultralow sulphur deficiency was determined from X-ray diffraction results as detailed in ref. 21. Additional information about the crystal growth and characterization is given in *SI Appendix*.

### Tunnel Diode Oscillator Measurements at High Pressures.

Quantum oscillations were measured via a contactless skin-depth probing technique based on the tunnel diode oscillator (TDO)—an inductor–capacitor oscillator sustained by a tunnel diode (36). The inductors in our experiments were 3 to 10 turn cylindrical coils with inner diameters of 80μm to 200μm wound with 12μm to 15μm insulated copper wires. The coils were mounted inside the <400 μm diameter sample spaces of BeCu or composite (38) gaskets of diamond/moissanite anvil cells. Samples of NiS_2_ were obtained by cleaving oriented single crystals into rectangular cuboids of 10μm to 100μm thickness and 50μm to 120μm length and width and were placed inside the coils. Our setup monitored the resonance frequency of the TDO. Changes in resistivity or magnetic susceptibility of NiS_2_ in the metallic state were detected as proportional shift in the resonance frequency, due to a change in the flux expulsion caused by the skin effect, which depends on resistivity and magnetic susceptibility. Wirings of the cryostats were optimized to allow the oscillator to operate at frequencies of up to 500 MHz. To ensure good hydrostaticity, we used a 4:1 methanol–ethanol mixture or 7474 Daphne oil (only at 55 kbar) as pressure-transmitting media. Pressure was determined via ruby fluorescence spectroscopy at low temperature. Quantum oscillations measurements were carried out at the NHMFL, Tallahassee, and at the HFML, Nijmegen, in top-loading ${}^{3}$He and dilution refrigeration cryostats with magnetic field strengths of up to 35T and at the superconducting high field facility in the Cavendish Laboratory, Cambridge, using a dilution refrigerator insert and fields of up to 18.4T. More details on the high-pressure tunnel diode oscillator measurements are provided in *SI Appendix*.

### Analysis of Quantum Oscillations.

The recorded TDO resonance frequency data contain the quantum oscillation signal on top of a large, slowly varying background as well as extrinsic artifacts, motivating post-processing such as piece-wise subtraction of a fitted background and band-pass filtering with a broad 4 kT to 8 kT pass band (e.g., Fig. 2), described in *SI Appendix*. The cross-sectional area $A_{k}$ associated with a cyclotron orbit in strong magnetic fields is determined via the Onsager formula (56) $A_{k}=\frac{2\pi e}{\hbar }F$, where e and ħ are the elementary charge and Planck’s constant, respectively, and F is the quantum oscillation frequency. The quasiparticle mass and the mean free path were obtained via full Lifshitz–Kosevich formula fitting (see *SI Appendix* for more details). The quasiparticle mass $m^{*}$ was additionally extracted by fitting the temperature (T) dependence of the quantum oscillation amplitude $\tilde{y}$ at a fixed magnetic field B with the Lifshitz–Kosevich expression $\tilde{y}=\alpha T{\left[\sinh\left(14.639\;{\text{TK}}^{-1}\frac{T}{B}\;\frac{m^{*}}{m_{e}}\right)\right]}^{-1}$, where α is a temperature-independent factor and $m_{e}$ is the bare electron mass.

## Supplementary Material

Appendix 01 (PDF)

## Data Availability

All data needed to evaluate the conclusions in the paper are present in the paper, the *SI Appendix*, and the Data Repository at the University of Cambridge and can be downloaded from DOI: https://doi.org/10.17863/CAM.100532.
