# Supplementary material for: Truncated mass divergence in a Mott metal
Source: Proc Natl Acad Sci U S A. 2023 Sep 11;120(38):e2301456120. doi: 10.1073/pnas.2301456120 (PMC10515144; doi:10.1073/pnas.2301456120)
Supplement: Supplementary file 1 — Appendix 01 (PDF) [file pnas.2301456120.sapp.pdf]

Supporting Information for

Truncated mass divergence in a Mott metal

Konstantin Semeniuk, Hui Chang, Jordan Baglo, Sven Friedemann, Stan Tozer, William A. Coniglio, Monika B. Gamża, Pascal Reiss, Patricia Alireza, Inge Leermakers, Alix McCollam, Audrey Grockowiak and F. Malte Grosche

Corresponding author: F. Malte Grosche.
E-mail: fmg12@cam.ac.uk

This PDF file includes:

Supporting text
Figs. S1 to S6
SI References

Supporting Information Text

1. Synthesis and characterization of NiS_2 single crystals

NiS_2 is prone to defects and therefore particular attention needs to be devoted to the growth and characterization of NiS_2 crystals for quantum oscillation measurements. A high degree of crystalline perfection is needed to observe quantum oscillatory phenomena. Whether a sample achieves the required long mean free paths is usually most conveniently assessed by measuring the residual resistivity in the low temperature limit, but screening samples in this way is far more laborious in the case of pressure-induced metal insulator transitions, because the metallic state can only be accessed by cumbersome high pressure transport measurements. Among the candidate materials considered for investigating the Mott metal-insulator transition under pressure, NiS_2 emerged as the most attractive choice because of previous reports that crystals with residual resistivities of less than $1\,\mu\Omega\,\mathrm{cm}$ in the pressure-induced metallic state could be grown (1). This demonstrates that it is in principle possible to achieve near-ideal stoichiometry and minimize the concentration of vacancies on the sulphur site to an acceptable level.

Single crystals of NiS_2 were grown with the tellurium-flux method as described earlier (2, 3). As outlined in Figure S1, polycrystalline $NiS_{2+\delta}$ of varying nominal sulphur excess δ was first prepared using a solid-state reaction, and single crystals were then grown from Te flux. Structural characterization of our crystals was carried out at room temperature using an Xcalibur E Single Crystal Diffractometer. Powder x-ray diffraction measurements were performed using a Bruker D2 Phaser diffractometer (Cu K-α radiation, 2θ interval of 3-80 degrees, step size of 0.018 degree, expose time of 4 hours) on powder prepared by grinding crystals with Si as an internal standard. Structure refinements based on both single crystal diffraction data and powder diffraction patterns were carried out using the Jana2006 program. X-ray diffraction measurements confirm that our crystals adopt the Pa-3 space group, the pyrite-type structure. Consistent results were obtained for several crystals across the batch. According to the single crystal X-ray diffraction study, our crystals are stoichiometric and have both Ni and S sites fully occupied (3). Refinements with initial values of occupancies set as 0.95 converged with negligible populations of vacancies $< 0.1\%$ for both crystallographic sites. This needs to be contrasted with previous studies on crystals grown using the vapor transport technique, for which $\simeq 2\%$ of S sites were found to be vacant (2, 4). Furthermore, our diffraction measurements performed on a number of crystals with typical dimensions of $0.1\,\mathrm{mm} - 0.2\,\mathrm{mm}$ showed mosaicities of only $0.4° - 0.5°$. Such low values of the mosaicity parameter indicate low long range disorder, which again is important for picking up quantum oscillations. Finally, there was no noticeable structured diffuse scattering in any of the diffraction images recorded from crystals used for the study, indicating the absence of clustered vacancy distributions that would lead to diffuse scattering visible as lines or other structures between the Bragg peaks. Also, no distinct systematic absence violations or superstructure reflections were observed. The successful refinement with full site occupation, the low mosaicities and the absence of structured diffuse scattering are all indicators of a high degree of perfection of the crystal lattice.

2. Tunnel-diode-oscillator measurements at high pressures

Quantum oscillations were measured via a contactless skin-depth probing technique based on the tunnel diode oscillator (TDO)—an inductor-capacitor oscillator sustained by a tunnel diode (5). The experiments were carried out at the NHMFL, Tallahassee, and at the HFML, Nijmegen, in top-loading ^{3}He and dilution refrigeration cryostats with magnetic field strengths of up to 35 T, and at the superconducting high-field facility in the Cavendish Laboratory, Cambridge, using a dilution refrigerator insert and fields of up to 18.4 T. For the measurements done at the Cavendish Laboratory and HFML we used the same setup, schematically shown in Figure S2, which will be used as a reference for the rest of this section. At the NHMFL we used a different TDO setup, which was provided by the local group, but had the same principle of operation (the setup was developed by Dr W. A. Coniglio).

A sample was placed inside a cylindrical sensor coil, which acted as the tank inductance. The coils were wound with $12\,\mu\mathrm{m} - 15\,\mu\mathrm{m}$ insulated copper wires, had 3-10 turns, and inner diameters of $80\,\mu\mathrm{m} - 200\,\mu\mathrm{m}$. The corresponding inductance (L) was of the order of tens of nH. The coils were mounted inside the $< 400\,\mu\mathrm{m}$ diameter sample spaces of BeCu or composite (6) gaskets of diamond/moissanite anvil cells. Samples of NiS_2 were obtained by cleaving oriented single crystals into rectangular cuboids of $10\,\mu\mathrm{m} - 100\,\mu\mathrm{m}$ thickness and $50\,\mu\mathrm{m} - 120\,\mu\mathrm{m}$ length and width, and were placed inside the coils. To ensure good hydrostaticity we used the 4:1 methanol-ethanol mix or 7474 Daphne oil (only at 55 kbar) as pressure-transmitting media. Pressure was determined via ruby fluorescence spectroscopy at low temperature.

The coil leads were fed out of the sample space and soldered to a section of flexible cryogenic coaxial copper cable at the gasket. This cable was connected to the tunnel diode and the tank capacitance (C) placed on a on a small piece of electrical circuit right outside the pressure cell. Keeping the distance between the coil and the tunnel diode as short as possible, as well as using a coaxial cable for connecting the two parts helped to minimize parasitic inductance and resistance, hence increasing sensitivity of the measurement. A board with the tunnel diode also contained a capacitor C_B, and a pair of resistors R_P and R_B. Use of higher current rating tunnel diodes generally made it easier to tune the oscillator into a stable regime, but caused more heating, limiting the lowest achievable temperature. The value of C was chosen based on the desired frequency of the oscillator, and was typically of the order of 10 pF. Resistance $R_P \simeq 100\,\Omega$ (which also accounts for the parasitic resistance) was required to make the circuit oscillate. Components R_B and C_B served the purpose of fine-tuning the oscillator.

Other parts of the oscillator circuit included the potential divider for biasing the tunnel diode as well as the signal demodulation and detection setup. Because of heat leakage and circuit optimisation considerations, part of the potential divider, containing resistors R_1 and R_2, was placed in the low-temperature region. Due to their substantial heat dissipation, these resistors could not be placed close to the pressure cell, and were therefore mounted on a separate board, connected to the

tunnel diode board with a coaxial copper cable and thermally anchored to the cryostat at the 1-K pot. In order to further limit the heat leakage, the subsequent connection to the room-temperature part of the circuit was made with a highly resistive stainless steel coaxial cable. With the described wiring, we could operate the oscillator at frequencies of up to 500 MHz.

The remainder of the biasing circuit was located at room temperature and contained the voltage source, resistors R_3, R_4, and R_5 of the potential divider, as well as capacitors C_1 and C_2, which acted as low-pass filters for the voltage source. The AC part of the signal was admitted to the demodulation and detection circuit via the capacitor C_3. The values of the aforementioned resistances and capacitances were typically slightly different for each experiment in order to optimize the setup for different cryostats and tunnel diodes. As an example, the following parameters were used for one of the configurations: $R_1 = 60\,\Omega$, $R_2 = 205\,\Omega$, $R_3 = 1\,\mathrm{k}\Omega$, $R_4 = 5\,\mathrm{k}\Omega$, $R_5 = 1.8\,\mathrm{k}\Omega$, $R_B = 51\,\Omega$, $R_P = 102\,\Omega$, $C_1 = 110\,\mathrm{nF}$, $C_2 = 110\,\mathrm{nF}$, $C_3 = 1\,\mathrm{nF}$, $C_B = 1\,\mathrm{nF}$, and $C = 12\,\mathrm{pF}$.

The raw AC signal entering the detection circuit was typically too weak and noisy to be directly fed into a frequency counter. The signal was first amplified with a powered +24 dB amplifier, and then shifted down in frequency from hundreds down to several tens of MHz via heterodyne mixing with a fixed frequency signal produced by a local oscillator. This allowed using passive band-pass filters with a narrower pass band in order to reduce the noise level. Depending on the resultant strength and quality of the signal, it could be amplified once again, down-mixed further to about 10 MHz and cleaned up with a low-pass filter. Finally, a frequency counter was used for tracking the frequency of the oscillator.

Our setup monitored the resonance frequency of the TDO. Changes in resistivity or magnetic susceptibility of NiS_2 in the metallic state were detected as a proportional shift in the resonance frequency, due to a change in the flux expulsion caused by the skin effect.

3. Quantum oscillation signatures of antiferromagnetic order in high pressure NiS_2

At pressures $p < 50\,\mathrm{kbar}$, the quantum oscillations signal at $F_0 \simeq 6\,\mathrm{kT}$ splits into two or even three closely-spaced frequencies (Figure 3b). Antiferromagnetic order is known to persist in NiS_2 well into the metallic part of the phase diagram (Figure 1). Neutron scattering data for $Ni(S/Se)_2$ (7), suggests non-collinear magnetic order with a magnetic unit cell that is identical to the crystallographic unit cell. Our DFT calculations within such an ordered state produce a modified FS geometry (Figure S3, see also (8)), in which the single large hole pocket splits into two pockets consistent with the frequency splitting at intermediate pressures reported above. In this regime, we obtain mass estimates from combined Lifshitz-Kosevich (LK) fits in which the masses for all the frequencies are constrained to be the same.

A number of reasons could be considered for why the spherical pocket has not yet been observed at 50 kbar and above: (i) the critical pressure at which antiferromagnetic order disappears might be lower in our crystals than what has previously been reported (e.g. (1, 9)), for instance due to the improved hydrostatic conditions evident from the observation of quantum oscillations in our experiment; (ii) the high magnetic fields at which quantum oscillations are measured could affect the magnetic order or entirely remove it, the more so as the magnetic quantum phase transition is approached with increasing pressure; (iii) the pressure dependence of the Fermi surface geometry could differ from the *ab initio* results in (8), for instance if the magnetic structure at high pressure is not as is currently deduced from ambient-pressure measurements on $NiS_{2-x}Se_x$. We note that the calculations in (8) give a critical pressure for antiferromagnetism of 200 kbar, whereas in experiments magnetic order is already suppressed at about 75 kbar, 45 kbar above the MIT; (iv) the larger curvature associated with the more spherical Fermi surface pocket or maybe a larger effective mass might decrease its signature in quantum oscillation measurements, causing it to remain undetected. None of these possibilities can at present be ruled out, and significant further work will be required to resolve the consequences of itinerant antiferromagnetism for the Fermi surface structure in high pressure NiS_2. This does not, however, affect the robust results of our study regarding the evolution of carrier mass with pressure.

4. Pressure dependence of lattice parameters of NiS_2

We investigated how the lattice parameters of NiS_2 evolved with pressure via powder X-ray diffraction measurements. The results are displayed in Figure S4. We found that in both insulating and metallic states the lattice constant decreased linearly with pressure with the same slope (up to the experimental uncertainty), up until the highest achieved pressure of 6.4 GPa. The data, however, also indicated that there was an additional 0.01 Å drop in the lattice constant, happening at some point between 2.8 GPa and 4.7 GPa. This observation is consistent with an earlier crystallographic study of NiS_2 (10), which revealed that the Mott transition in the system is accompanied by a structural change. At lower pressures, the crystal was found to have a cubic symmetry, which was reported to change to a weakly monoclinic one in the metallic state (the reported lattice parameters at 38.6 kbar were $a = 5.5748(9)$ Å, $b = c = 5.5853(6)$ Å, and $\beta = 89.949(1)°$). Our experiment did not resolve the deviation from cubic symmetry at higher pressures reported in (10), possibly because of limitations of the apparatus.

5. Analysis of quantum oscillations

Here we describe the procedure for analysing the magnetic field dependence of the TDO frequency and determining parameters related to the electronic structure of NiS_2.

First, a number of corrections had to be made to the recorded signal. In each of our experiments, magnetic field strength was deduced from the current flowing in the magnet coils. For the measurements conducted with resistive magnets at NHMFL and HFML, noise in the current readout resulted in slight fluctuations in the reported magnetic field values. In some experiments these fluctuations reached a sizeable fraction of a quantum oscillation period, obscuring the oscillations in the raw data. This

problem was solved by smoothing the raw magnetic field strength values against time using a moving average filter. This procedure greatly improved visibility of quantum oscillations, which in turn implied that the noise was not intrinsic to the current, but was rather a measurement artefact.

The next correction was related to the fact that position of the sample during the measurements did not perfectly coincide with the centre of the magnetic field profile. This meant that the field strength experienced by the sample (B_{true}) was lower than the nominal field strength value (B_{read}). The former can be related to the latter via a multiplicative factor slightly less than 1. Another issue was a time delay between the acquisitions of the TDO frequency and magnetic field strength readings, meaning that B_{read} was additionally shifted with respect to B_{true}, proportionally to the field sweep rate. The relationship between the true and recorded magnetic field strength values can be captured with the following formula:

$$B_{\mathrm{true}} = \alpha \left(B_{\mathrm{read}} + \tau \frac{dB_{\mathrm{read}}}{dt} \right) \ , \qquad [1]$$

where α is a scaling factor due to the mispositioning of the sample, τ is a time delay between acquiring the values of of B_{read} and the TDO frequency for a given data point, and dB_{read}/dt is the field sweep rate. The TDO happened to be sensitive to nuclear magnetic resonances (NMR) of certain chemical elements abundant in the sample space, which provided an intrinsic way of determining the values of α and τ. The resonances manifested as sharp peaks in the signal. Given an electromagnetic field oscillating at a frequency ν, the resonance for a particular element occurs when the field strength is equal to ν/γ, where γ is the gyromagnetic ratio of that element. The NMR peaks relevant for our experiments originated from ^{1}H in the methanol-ethanol pressure medium ($\gamma = 42.5775\,\mathrm{MHz\,T^{-1}}$) as well as ^{63}Cu and ^{65}Cu. Rather than using bare gyromagnetic ratios of atomic copper, we calculated the corresponding values for the bulk metal forms of the isotopes. Using information in the literature (11–14), we obtained $11.3122(3)\,\mathrm{MHz\,T^{-1}}$ and $12.1177(4)\,\mathrm{MHz\,T^{-1}}$ for ^{63}Cu and ^{65}Cu, respectively. For each valid NMR peak, we determined the resonance field B_{read} by fitting the signal with the absorption-dispersion lineshape (15) and noted down the corresponding values of $B_{\mathrm{true}} = \nu/\gamma$ and dB_{read}/dt. After all the NMR peaks were processed, we obtained α and τ by fitting the expression (1) to the recorded values of B_{true}, B_{read}, and dB_{read}/dt. We then used the same formula to convert B_{read} to B_{true} in all the sweeps. For this approach to work well, dB_{read}/dt had be sufficiently varied across the processed sweeps. When this was not the case, we determined τ by finding the value that minimized the relative phase shifts of quantum oscillations recorded at different sweep rates.

High sensitivity of the TDO setup made the signal very susceptible to extrinsic influence, such as movement of a cryostat probe or vibration of cables. These effects resulted in artifacts in the signal such as increased noise, sudden jumps, or rapid drifts. Discontinuities were eliminated by offsetting the TDO frequency data. When possible, random noise was reduced by averaging multiple sweeps performed at the same temperature. Next, background subtraction was performed in order to isolate the oscillatory part of the signal. For presentation purposes such as the preparation of Fig. 2a in the main text, the field corrected data could further be subjected to a band-pass filter with a broad pass band (Figure S5). Lifshitz-Kosevich fits for the purpose of extracting parameters such as mass and mean free path have been carried out on unfiltered data.

Our TDO setup effectively probed changes in the skin depth of the sample, which affected the oscillator frequency via change of inductance of the sensing coil. The skin depth (δ) depends on electrical resistivity (ρ) and magnetic permeability (μ), both of which exhibit quantum oscillations. Moreover, small relative changes in the two quantities ($\Delta\rho/\rho$ and $\Delta\mu/\mu$) have an equally strong effect on δ. It is therefore not obvious whether the observed quantum oscillations should be treated as the Shubnikov-de-Haas (SdH) effect, de-Haas-van-Alphen (dHvA) effect, or a mixture of both. Starting with the Lifshitz-Kosevich (LK) formula for quantum oscillations in magnetisation (16), an expression for quantum oscillations in magnetic susceptibility (χ_{osc}) can be obtained by differentiating the formula with respect to magnetic field. When frequency of quantum oscillations (F) is much larger than the magnetic field strength (B), which was the case in our experiments, χ_{osc} is dominated by a single term, which has the amplitude (ignoring the phase smearing factors):

$$\chi_{\mathrm{osc},0} = \left(\frac{e}{\hbar} \right)^{\frac{3}{2}} \frac{\mu_0}{\sqrt{2\pi}} \frac{k_{\mathrm{B}} T}{|A''_{\parallel}|^{\frac{1}{2}}} \frac{F^2}{B^{\frac{5}{2}}} \ , \qquad [2]$$

where e, μ_0, and $\hbar$ denote the usual fundamental constants, and $A''_{\parallel}$ is the second derivative of the Fermi surface cross section area in the plane normal to the field with respect to the wavevector in the field direction, evaluated at the extremal cross section. Since relative permeability is close to unity, the amplitude of $\Delta\mu/\mu$ is approximately equal to $\chi_{\mathrm{osc},0}$. In the case of SdH oscillations, it can be shown that the corresponding amplitude of $\Delta\rho/\rho$ is roughly equal to $\sqrt{B/2F}/|A''_{\parallel}|^{\frac{1}{2}}$ (16). For $F = 6000\,\mathrm{T}$, $B = 25\,\mathrm{T}$, and $T = 1\,\mathrm{K}$, $\Delta\rho/\rho$ is about 24 times larger than $\Delta\mu/\mu$. Based on this, we assumed that quantum oscillations manifesting in the TDO frequency (Δf_{TDO}) represented the SdH effect, and since $\Delta\rho/\rho$ was small, Δf_{TDO} was approximately linear in it, and could therefore be described with the corresponding LK formula (16):

$$\Delta f_{\mathrm{TDO}} = \frac{CT}{\sqrt{B}} \frac{\exp\left(-\beta T_{\mathrm{D}} m^*/B\right)}{\sinh\left(-\beta T m^*/B\right)} \cos\left(2\pi F/B + \phi\right) \ , \qquad [3]$$

where C is a field- and temperature-independent multiplier, T_{D} is the Dingle temperature, m^* is the effective quasiparticle mass (in units of the free electron mass), ϕ is the phase shift of the oscillations, and β is a constant equal to $14.639\,\mathrm{T\,K^{-1}}$. The exponential multiplier accounts for the effect of disorder on quantum oscillations, which is quantified by the Dingle temperature, defined as

$$T_{\mathrm{D}} = \hbar / (2\pi k_{\mathrm{B}} \tau) \ , \qquad [4]$$

where τ is the mean scattering time. If the values of T_{D}, m^*, and F are known, then using the Onsager relation

$$\frac{1}{F} = \frac{2\pi e}{\hbar A_{\parallel}} \ , \qquad [5]$$

and assuming a spherical Fermi surface, one can estimate the mean free path l:

$$l = \frac{\sqrt{2e\hbar^3 F}}{2\pi m^* k_{\mathrm{B}} T_{\mathrm{D}}} \ . \qquad [6]$$

In order to extract the relevant physical quantities from the data, we fitted the processed, background-subtracted field sweeps with the SdH LK formula, using the least-squares method, with C, T_{D}, m^*, F, and ϕ as the fitting parameters. Since measurements were conducted at temperatures below 2 K, it is reasonable to assume that the mean free path and consequently T_{D} are independent of temperature for a given pressure. We also assumed no implicit dependence of Δf_{TDO} on B. It is important to note, that for a single field sweep at a fixed temperature, parameters m^* and T_{D} are correlated, i.e. their variation has a similar effect on the function, making the fitting underconstrained. To circumvent this problem, we fitted the LK formula globally to all the sweeps at a particular pressure, forcing the listed fitting parameters to be fixed at all temperatures. Since T_{D} does not affect the relative temperature dependence of f_{TDO}, and m^* does, the global fitting allows to decouple the two parameters. The result of applying this procedure to one of the pressure points is illustrated in Figure S6. In order to additionally verify the results of the fitting, we also used an alternative method of determining the effective mass, by considering the change of quantum oscillation amplitude ($\tilde{y}$) with temperature at fixed magnetic field predicted by the LK formula: $\tilde{y} = \alpha T \left[\sinh\left(14.639\,\mathrm{TK}^{-1} \frac{T}{B} \frac{m^*}{m_e}\right)\right]^{-1}$, where α is a temperature-independent factor and m_e is the bare electron mass. Figure 2b of the main text shows an application of this method. The two approaches resulted in consistent effective mass values.

For certain pressures, quantum oscillations exhibited beating, suggesting the presence of multiple closely spaced frequencies (shown in Figure S6). In these cases, the fitted LK formula consisted of multiple terms, and each was allowed to have a separate set of values for F, C, and ϕ. We constrained the values of T_{D} and m^* to not vary across the different frequency components in order to keep the number of fitting parameters low enough. The resultant values of T_{D} and m^* can therefore be considered as effective average values across all the quantum oscillation orbits present. Additional frequency terms were added, up to a maximum of three terms in total, as long as it improved the fit. The spread of frequencies across the different terms was always less than 5%.

While the uncertainty in F was negligible, thanks to each field sweep spanning tens of oscillation periods and more, the values of m^* were far less precise, primarily due to an uncertainty in temperature. Error bars for m^* were estimated by fitting the LK formula repeatedly for a 100 times, with the temperature values for each fit randomly and uniformly sampled from an interval corresponding to the uncertainty in temperature (due to fluctuations and a drift). The resultant spread of m^* at each pressure determined the size of the respective error bars.

6. Conversion of quantum oscillation mean free path to residual resistivity, effective medium theory

A. Residual resistivity. The QO mean free path, as determined from the Dingle analysis outlined above, is compared to the residual residual resistivity ρ_0 in Figure 3d. The conversion uses the Drude result derived in the supplementary material to (17), which uses the DFT electronic density of states per unit volume at the Fermi level $g(E_F)$ and the DFT plasma frequency Ω_p:

$$\ell = \left(\frac{3}{\epsilon_0 e^2 g(E_F) \Omega_p^2}\right)^{1/2} \frac{1}{\rho_0} \ . \qquad [7]$$

The plasma frequency Ω_p is obtained from the direction-dependent plasma frequencies, which are calculated from Wien2K reults by the OPTIC package (18), using the relation

$$\Omega_x^2 = \frac{e^2}{4\pi\epsilon_0} \frac{1}{\pi^2 m^2} \sum_{n,\mathbf{k}} p_{x;n,\mathbf{k}}^2 \delta(\epsilon_{n,\mathbf{k}} - \epsilon_F) \ , \qquad [8]$$

and likewise for the other principal axes y and z. Here, $\mathbf{p}_{n,\mathbf{k}}$ is the momentum expectation value for states in band n with crystal momentum $\mathbf{k}$. The overall plasma frequency is then estimated by averaging the squared frequencies: $(\Omega_p^{(0)})^2 = \frac{1}{3}(\Omega_x^2 + \Omega_y^2 + \Omega_z^2)$, but as NiS_2 is cubic, all three direction-dependent plasma frequencies are the same and are equal to Ω_p, for which we obtain $\Omega_p = 3.27\,\mathrm{eV}$. Our Wien2K density of states is $g(E_F) = 30.88\,/\mathrm{Ryd}$ per formula unit of NiS_2, and we take the volume per formula unit as $V_0 = 43.67\,\text{Å}^3$. With these parameters, Eqn. 7 translates to the conversion $\rho_0 \simeq 1270\,\text{Å}\,\mu\Omega\,\mathrm{cm}/\ell$. The blue markers in Figure 3d of the main paper have been placed according to this conversion.

B. Effective medium theory. Our measured pressure dependence of the low-temperature resistivity provides support for the formation of an inhomogenous metallic-insulating phase across the MIT in NiS_2. In such a phase, transport properties can depend on microscopic details such as the formation of domains, conductive planes or regular three-dimensional networks. Since such structural evidence is not available for NiS_2 at the MIT, we employ the simplest model based on the effective medium theory (19, 20). In this model, it is assumed that randomly located, spherical metallic islands form inside the insulating bulk (or vice versa) with an average metallic ratio $0 \leq x \leq 1$. As shown in Figure 3d, we model the effective resistivity $\rho_{\text{eff}}(x)$ as a function of the continuously varying implicit variable $x(p)$ across the MIT, with $x = 0$ in the most insulating, low-pressure phase and $x = 1$ in the metallic, high-pressure phase. At intermediate x, the effective resistivity is given as

$$\sum_i w_i \frac{\rho_{\text{eff}} - \rho_i}{\rho_{\text{eff}} + (d-1)\rho_i} = 0 \qquad [9]$$

where $w_1 = x$, $\rho_1 = \rho_{\text{metallic}}$ describe the metallic fraction and its resistivity, whereas $w_2 = 1 - x$, $\rho_2 = \rho_{\text{insulating}}$ correspond to the insulating fraction, and the dimensionality $d = 3$. The red line in Figure 3d shows the result of solving Equation 9 for ρ_{eff} and fitting ρ_{eff} to the measured $\rho(p)$, with ρ_{metallic}, $\rho_{\text{insulating}}$ and $x(p) \sim p$ being fitting parameters.

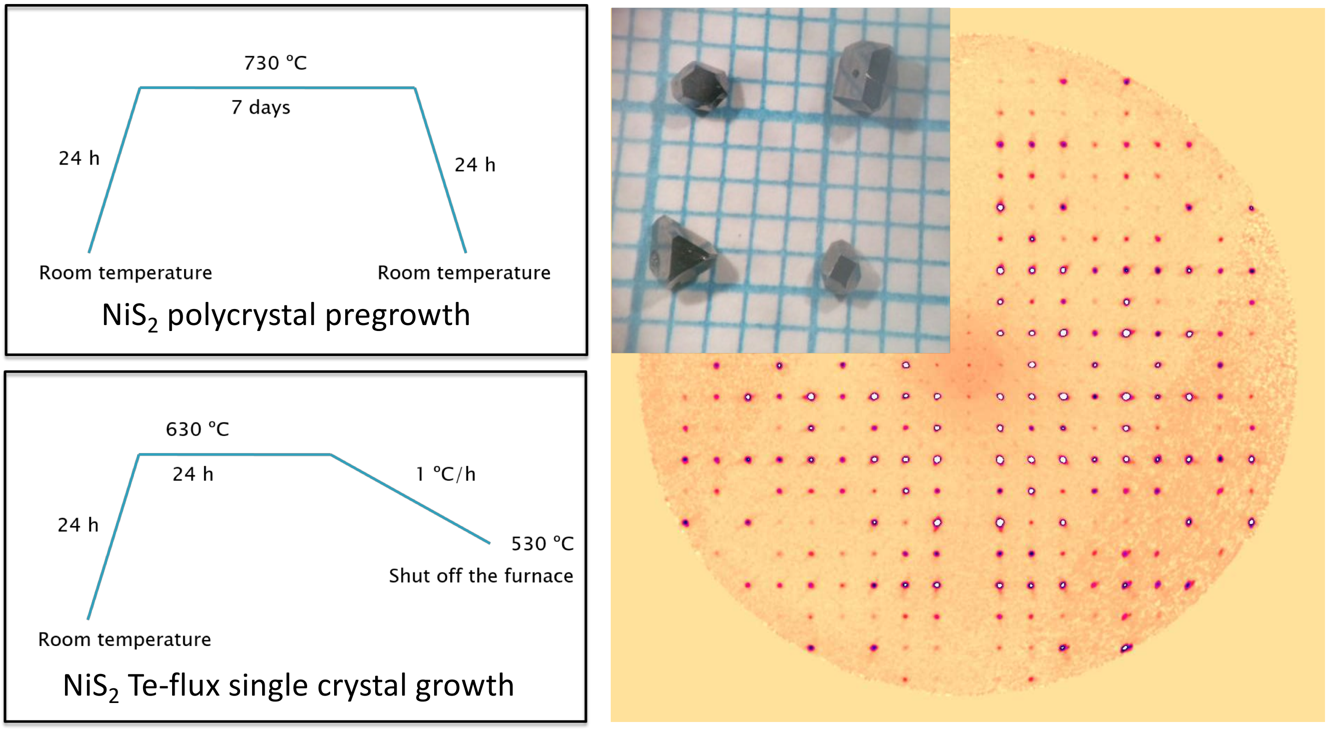

Fig. S1. NiS_2 growth and characterization, following the approach described in (2) and (3). (top left) NiS_2 polycrystals are prepared using a conventional solid state reaction. (bottom left) These polycrystals are used as the starting material for Te-flux growth of single crystals. (right) Millimetre-sized single crystals are extracted (inset) and characterized by single-crystal X-ray diffraction. Resistivity measurements under pressure (3) have demonstrated that these crystals achieve residual resistivities $< 1\,\mu\Omega\,\mathrm{cm}$ in the pressure-induced metallic state.

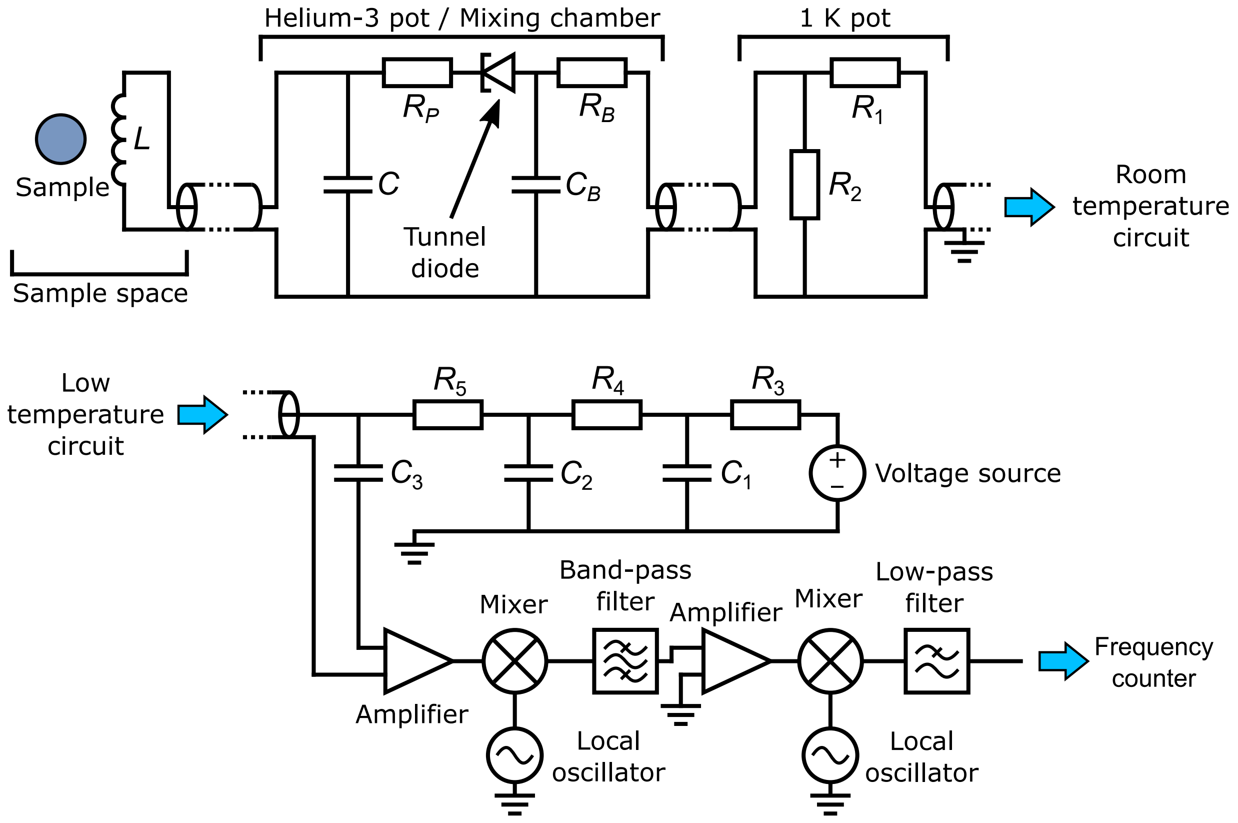

Fig. S2. Full circuit diagram of the tunnel diode oscillator setup for contactless skin depth measurements. The low-temperature and room-temperature parts of the circuit are shown, respectively, in the top and bottom parts of the figure.

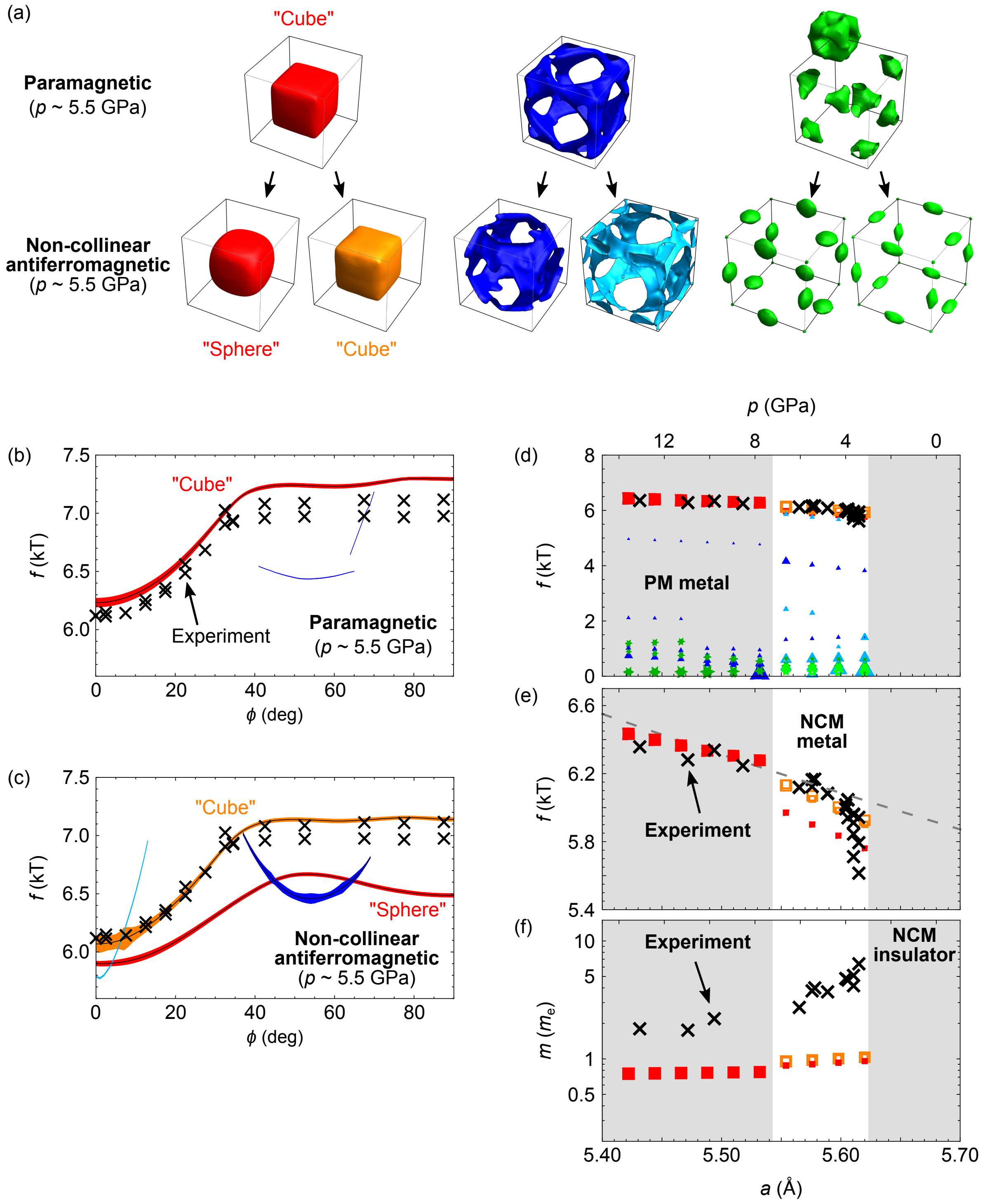

Fig. S3. DFT predictions for metallized NiS_2: (a) Fermi surface topology for the paramagnetic (PM) and non-collinear antiferromagnetic (NCM) phases, under a pressure of $p \approx 5.5$ GPa, well inside the metallic phase. The NCM order leads to a splitting of the paramagnetic Fermi surface sheets as shown. Notably, the approximate cubic shape of one Fermi surface sheet is retained. (b,c) Predicted (lines) and observed (crosses) angular dependence of quantum oscillation frequencies under a pressure of $p \approx 5.5$ GPa, assuming either a paramagnetic phase (b) or the NCM phase (c). (d-f) Pressure dependence of observed and predicted quantum oscillation frequencies and effective masses. The different pressure ranges highlighted by a shaded background mark the experimentally paramagnetic (PM) metallic phase, the NCM metallic phase, and the experimentally insulating phase (9). Panel (d) shows all predicted frequencies, while panels (e) and (f) show predictions for the cubic / spherical Fermi surface sheets only, see panel (a). The dashed line in (e) is a guide-to-the-eye assuming $f \sim 1/a^2$ corresponding to a constant charge carrier number as a function of lattice constant a. For panels (b)-(f), the symbol sizes and line thicknesses represent the predicted quantum oscillation signal strength as deduced from the curvature factor. The color coding corresponds to the Fermi surface sheets as shown in (a).

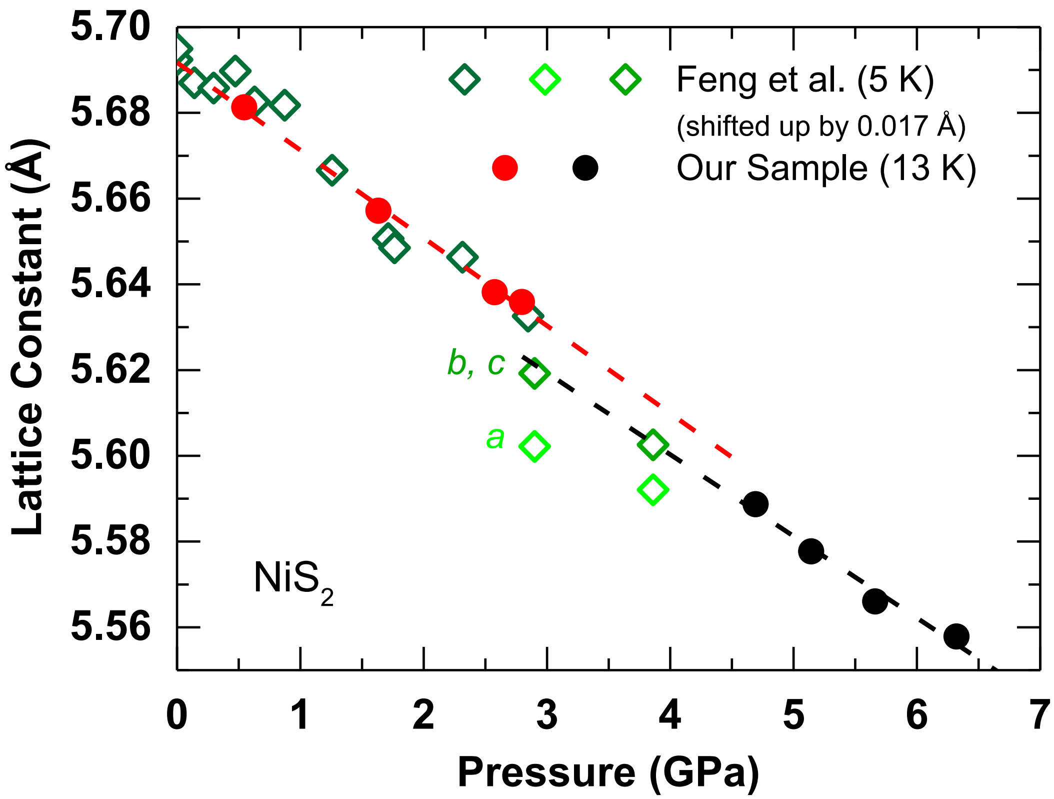

Fig. S4. High-pressure powder X-ray diffraction studies of NiS_2: Reduction of the lattice constant with pressure has the same linear trend (dashed lines) for both insulating and metallic states, but there appears to be a discontinuous drop of about 0.01 Å at the phase boundary. The same drop has been observed in an earlier study by Feng et al. (10), which also showed the change of symmetry from cubic to weakly monoclinic.

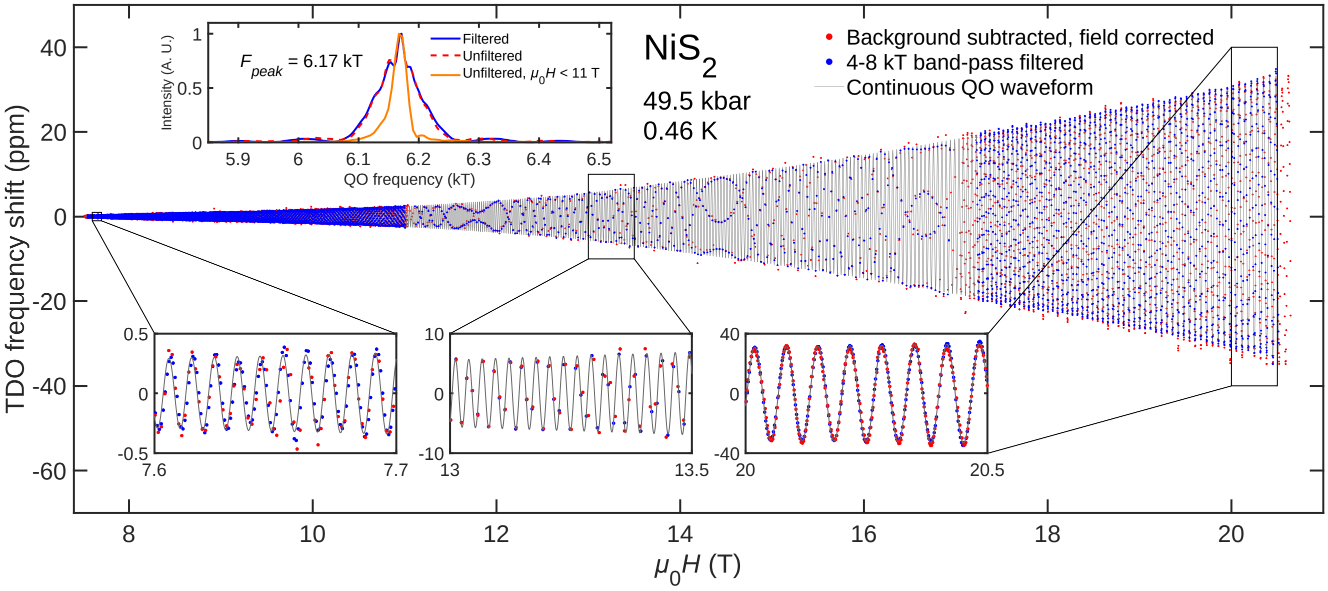

Fig. S5. Effect of band-pass filtering. (main panel) A 4-8 kT passband has been used on a scan at about $50\,\mathrm{kbar}$, producing the data shown in Fig. 2a of the main text. (upper inset) This causes no changes to the power spectrum in the range of interest, nor does it materially affect the high field data (right inset), but it filters out some outliers at low fields (bottom left and central inset).

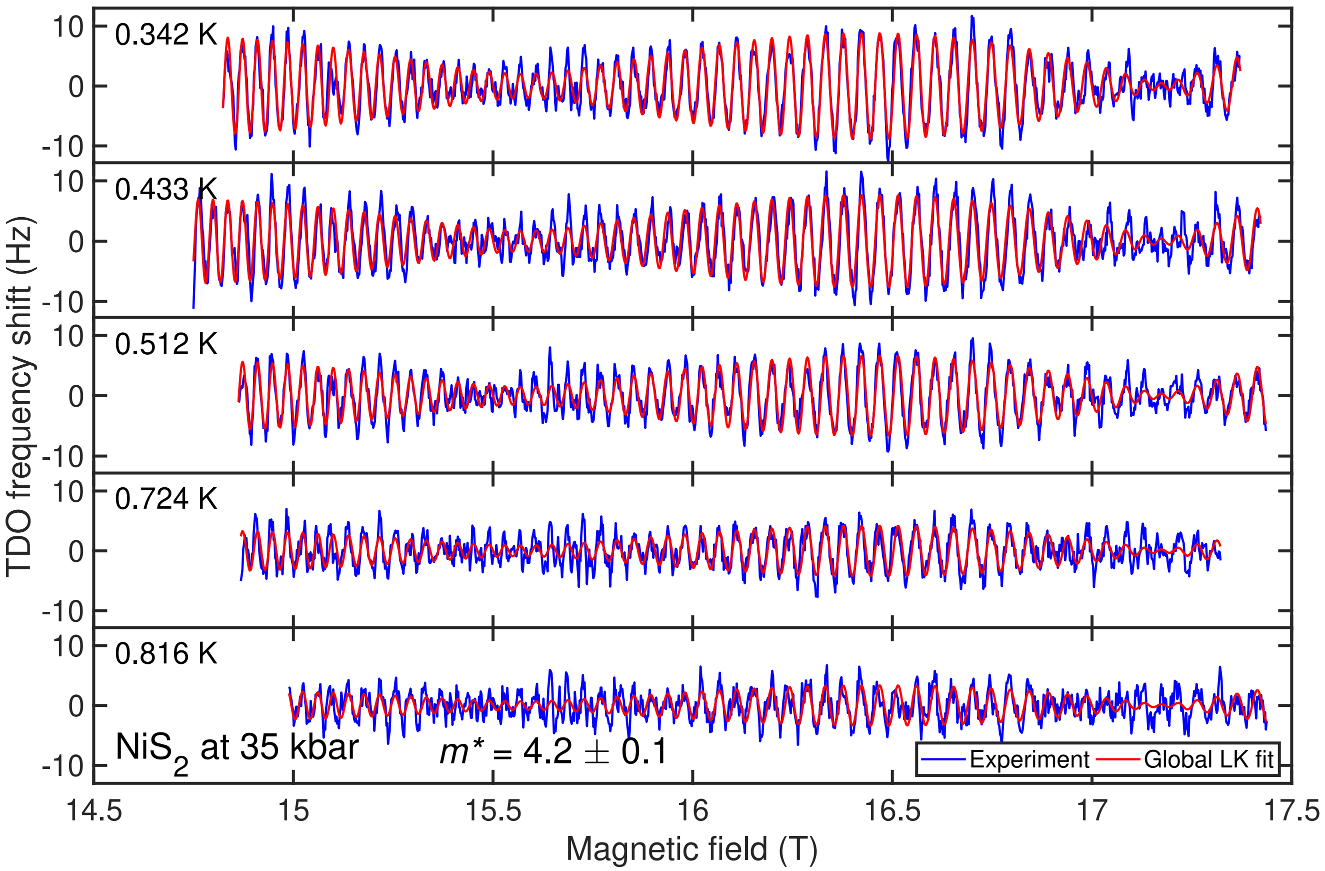

Fig. S6. Modelling quantum oscillations in NiS_2: Background-subtracted magnetic field dependence of TDO frequency at multiple temperatures for NiS_2 at $35\,\mathrm{kbar}$. The data are fitted with the Lifshitz-Kosevich formula for Shubnikov-de-Haas oscillations. The fitting was performed simultaneously for all the sweeps at different temperatures, using a temperature-independent set of fitting parameters. For this particular set of data, quantum oscillations exhibit beating, which was captured with the Lifshitz-Kosevich formula with three terms.

References

1. N Takeshita, et al., Quantum criticality and disorder in the antiferromagnetic critical point of NiS_2 pyrite. *arXiv* pp. cond–mat/0704.0591 (2007).
2. X Yao, JM Honig, Growth of nickel dichalcogenides crystals with pyrite structure from tellurium melts [NiS_2, $NiS_{2\text{-}x}Se_x$ (x $\leq$ 0.7)]. *Mater. Res. Bull.* **29**, 709–716 (1994).
3. S Friedemann, et al., Large Fermi Surface of Heavy Electrons at the Border of Mott Insulating State in NiS_2. *Sci. Rep.* **6**, 25335 (2016).
4. P Kwizera, MS Dresselhaus, D Adler, Electrical properties of Ni S 2 ? x Se x. *Phys. Rev. B* **21**, 2328–2335 (1980).
5. CT Van Degrift, Tunnel diode oscillator for 0.001 ppm measurements at low temperatures. *Rev. Sci. Instruments* **46**, 599–607 (1975).
6. DE Graf, RL Stillwell, KM Purcell, SW Tozer, Nonmetallic gasket and miniature plastic turnbuckle diamond anvil cell for pulsed magnetic field studies at cryogenic temperatures. *High Press. Res.* **31**, 533–543 (2011).
7. S Yano, et al., Magnetic structure of $NiS_{2\text{-}x}Se_x$. *Phys. Rev. B* **93** (2016).
8. P Reiss, S Friedemann, FM Grosche, *Ab initio* electronic structure of metallized NiS 2 in the noncollinear magnetic phase. *Phys. Rev. B* **106**, 205131 (2022).
9. PG Niklowitz, et al., Unconventional resistivity at the border of metallic antiferromagnetism in NiS_2. *Phys. Rev. B* **77** (2008).
10. Y Feng, R Jaramillo, A Banerjee, JM Honig, TF Rosenbaum, Magnetism, structure, and charge correlation at a pressure-induced Mott-Hubbard insulator-metal transition. *Phys. Rev. B* **83** (2011).
11. ER Andrew, Developments in the motional narrowing of the NMR spectra of solids - microscopic and macroscopic. *Pure Appl. Chem.* **32**, 41–50 (1972).
12. K Kitagawa, et al., Space efficient opposed-anvil high-pressure cell and its application to optical and NMR measurements up to 9 GPa. *J. Phys. Soc. Jpn.* **79**, 024001 (2010).
13. O Lutz, H Oehler, P Kroneck, ^{63}Cu and ^{65}Cu Fourier transform nuclear magnetic resonance studies. *Z. Für Phys. At. Nucl.* **288**, 17–21 (1978).
14. O Lutz, H Oehler, P Kroneck, Chemical shifts and coupling constants in copper (i)-compounds by ^{63}Cu and ^{65}Cu FT-NMR studies. *Z. Für Naturforschung A* **33**, 1021–1024 (1978).
15. J Keeler, *Understanding NMR Spectroscopy.* (Wiley, Chichester, England Hoboken, NJ), (2005).
16. D Shoenberg, *Magnetic Oscillations in Metals.* (Cambridge University Press, Cambridge), 1 edition edition, (1984).
17. J Chen, MB Gamża, K Semeniuk, FM Grosche, Composition dependence of bulk superconductivity in YFe_2Ge_2. *Phys. Rev. B* **99**, 020501(R) (2019).
18. C Ambrosch-Draxl, JO Sofo, Linear optical properties of solids within the full-potential linearized augmented planewave method. *Comput. Phys. Commun.* **175**, 1–14 (2006).
19. DAG Bruggeman, Berechnung verschiedener physikalischer Konstanten von heterogenen Substanzen. I. Dielektrizitätskonstanten und Leitfähigkeiten der Mischkörper aus isotropen Substanzen. *Ann. Phys.* **24**, 636 (1935).
20. S Kirkpatrick, Percolation and Conduction. *Rev. Mod. Phys.* **45**, 574–588 (1973).
